# Assessment of Nonnucleoside Inhibitors Binding to HIV-1 Reverse Transcriptase Using HYDE Scoring

**DOI:** 10.3390/ph12020064

**Published:** 2019-04-24

**Authors:** Agata Paneth, Wojciech Płonka, Piotr Paneth

**Affiliations:** 1 Faculty of Pharmacy with Medical Analytics Division, Medical University of Lublin, Chodźki 4a, 20-093, Lublin, Poland; agata.paneth@umlub.pl; 2 FQS-Fujitsu Poland, Parkowa 11, 33-332 Kraków, Poland; w.plonka@fqs.pl; 3 Institute of Applied Radiation Chemistry, Lodz University of Technology, Żeromskiego 116, 90-924 Łódź, Poland

**Keywords:** HIV-1 reverse transcriptase, docking, HYDE, QSAR

## Abstract

In this study, 48 inhibitors were docked to 107 allosteric centers of human immunodeficiency virus 1 (HIV-1) reverse transcriptase from the Protein Data Bank (PDB). Based on the average binding scores, quantitative structure-activity relationship (QSAR) equations were constructed in order to elucidate directions of further development in the design of inhibitors. Such developments, informed by structural data, must have a focus on activity against mutated forms of the enzyme, which are the cause of the emergence of multidrug-resistant viral strains. Docking studies employed the HYDE scoring function. Two types of QSARs have been considered: One based on topological descriptors and the other on structural fragments of the inhibitors. Both methods gave similar results, indicating substructures favoring binding to mutated forms of the enzyme.

## 1. Introduction

Approximately 77 million people have become infected with human immunodeficiency virus-1 (HIV-1) since the first cases were reported in 1981 [1]. By the end of 2016, the World Health Organization estimated that approximately 36.7 million people were currently living with HIV-1 infection, including 1.8 million newly infected people in 2016 [2]. Over the past two decades, significant advances have been mounted to address the epidemic and significant progress has been made. However, because of toxicity, the rapid emergence of resistance, detrimental side effects caused by long-term drug treatment and issues associated with drug tolerability, there remains a need for new antiviral agents [3,4].

Due to well-characterized mechanisms of action and abundant structure information, reverse transcriptase (RT)—one of three essential enzymes encoded in the HIV-1 genome—represents a successful target for chemotherapeutic intervention [5,6]. RT inhibitors may be divided into nucleoside/nucleotide RT inhibitors (NRTIs/NtRTIs) and non-nucleoside RT inhibitors (NNRTIs) [7]. These inhibitors are widely used in highly active antiretroviral therapy (HAART) regimens, owing to their potent activity, high selectivity, and favorable pharmacokinetics [8]. NNRTIs in particular act by disrupting the normal functions of RT via binding to the NNRTI binding pocket (NNIBP), close to the polymerase active site [7]. So far, six NNRTIs have been licensed for use [9]. Nevirapine, delavirdine, and efavirenz are the first-generation NNRTIs with high potency [10]. However, central nervous system side effects, hepatotoxicity, poor resistance profile and low genetic barriers for viral resistance are the major treatment-limiting factors in their clinical application [9,11]. In particular, single mutants K103N, Y181C and double mutation K103N/Y181C are prevalent in clinical HIV-1 isolates [12,13]. Even in naive patients, low frequencies of these mutant variants can lead to an increased risk of virologic failure [14].

In an effort to develop novel NNRTIs with improved antidrug resistance profiles, two other drugs—i.e., etravirine and rilpivirine—have been approved by the U.S. Food and Drug Administration (FDA) and European Union in 2008 and 2011, respectively [15]. As their mechanism of action is different from the first-generation NNRTIs, they are called the second-generation NNRTIs. Both of these drugs are diarylpyrimidine derivatives, the group of heterocycles that resembles pyrimidine nucleosides found in DNA [16]. Their conformational flexibility (along with the plasticity of the binding sites of RT) give them a very high potency and are reportedly less likely to generate resistance compared to other NNRTIs [17,18]. However, both etravirine and rilpivirine suffer from poor solubility. Etravirine is practically insoluble in water over physiological pH, leading to a daily dosage of 400 mg due to extensive formulation work, while rilpivirine is hardly dissolved in water, making it display an atypical absorption mechanism involving aggregates. Moreover, it was found that only about one-third of patients could retain full susceptibility to the two diarylpyrimidine drugs; for etravirine, 36.5%; for rilpivirine, 27.3% [19]. In addition, etravirine shows severe side-effects, such as peripheral neuropathy, skin rashes, and hepatotoxicity, and was even listed as a “dangerous drug” by the U.S. FDA in 2008. Finally, NNRTI resistance-associated mutations are still observed in patients receiving second-generation NNRTIs regimens [3,20]. Furthermore, when resistance to rilpivirine is selected after virologic failure, broad cross-resistance profiles against almost all of the NNRTI drugs is commonly observed [3,21,22,23,24,25,26,27,28,29,30]. Thus, it is still urgently needed to identify novel NNRTIs with high potency against resistance mutations, improved water solubility and favorable safety profiles.

We have addressed this problem recently using computational methods that included a quantitative structure-activity relationship (QSAR) model based on average binding scores of 48 inhibitors and structural information of 107 allosteric centers of HIV-1 reverse transcriptase. In the present contribution, we extend our studies to the newer scoring function HYDE that proved very successful in docking studies [31]. Additionally, in the quest for elucidation of directions of further development in the design of inhibitors for mutated forms of the enzyme, we constructed QSAR equations based on descriptors and substructures for the wild type enzyme and for its mutants.

## 2. Results and Discussion

**Figure 1:**
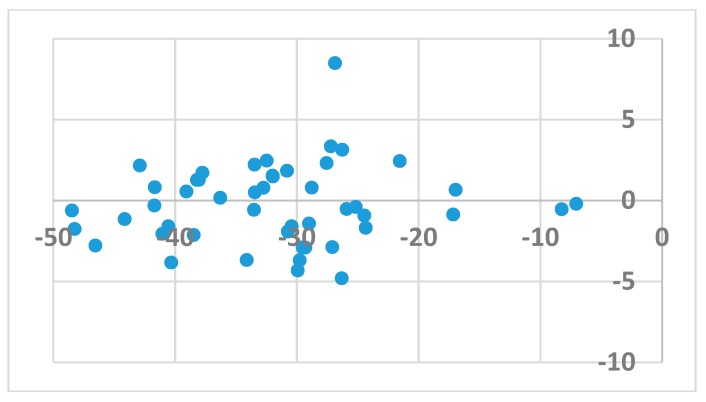
The signed difference in averaged HYDE score between binding to wild type enzyme vs. binding to mutants as a function of the binding score.

The procedure used in docking followed that previously reported for the FlexX scoring function [32]. In short, 48 ligands were docked to the allosteric cavity of 107 HIV-1 RT enzymes (available from the PDB [33]), and an average binding score for a given ligand was obtained separately for the wild type (wt) and mutated enzyme. Poses (Table 1) were inspected for correct orientation within the cavity. Average binding scores obtained for the wt and mutated enzymes were then compared. As illustrated in Figure 1, binding of either the wt enzyme or its mutated forms was generally random and did not correlate with the calculated binding energy. This observation indicates that activity against mutated forms of the enzyme is not governed by the strength of binding. A sole exception to the above observation is the result obtained for the DJZ ligand, which binds to the mutated forms of HIV-1 RT much stronger than to its wt structures.

**Figure 2:**
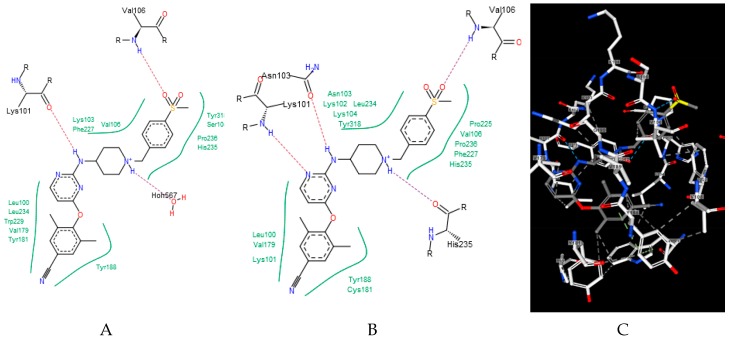
Binding of a DJZ ligand in allosteric pockets of (**A**) a wt native enzyme (3m8q) and (**B**) a K103N/Y181C mutant (Protein Data Bank [PDB] 3bgr). (**C**) A 3D representation of B.

Ligands that bind to the allosteric site impair enzyme action by a wedge mechanism; they hinder domain mobility, opening and closing access to the active site. Final allosteric site architecture is achieved upon binding of the ligand. This flexibility and the possible clash between protein and ligand was accounted for by using a large overlap volume (100 Å^3^). Lack of systematic differences between binding to the wt versus the mutated enzyme indicates that activity against mutants is connected with the structural features of the ligand, rather than the energy of their binding. Interactions within the allosteric site are mostly associated with van der Waals forces and, to a lesser extent, hydrogen bonding [33]. Ligand H18 exhibited the strongest binding to all forms of the enzyme, however, as mentioned above, it is a DJZ ligand that shows the largest change in binding energy when moving from the wt to the mutated enzymes. Its success seems to come from hydrogen bonding to lysine 101 rather than frequently mutated lysine 103. Furthermore, its orientation within the pocket is improved by a stronger and stiffer hydrogen bond from piperidine to histidine, compared to a water molecule, and an additional hydrogen bond to the pyrimidine ring (compare panels of Figure 2).

The results obtained from docking were used in the QSAR studies. In order to understand the very general structural features affecting the binding affinity, two types of QSAR models were used. The first one was based on descriptors that had clear “chemical interpretation”, giving suggestions for composition and physico-chemical properties in the new compound design. In this approach, we used the algorithms and the library of descriptors implemented in the SCIGRESS Suite software [34].

The best relationships were identified for both enzyme types. In both cases, they included the same four descriptors out of 383 evaluated descriptors:

HYDE score (wt) = 2234.2083 × carbonyl count/MW + 0.0295 × hydrophobic dipole^2^ + 8.8541 × ln(Nitrogen count) + 1006.2207 × 1.0/all bond count − 68.5158 (1)

and

HYDE score (mutants) = 2130.1116 × carbonyl count/MW + 0.0232 × hydrophobic dipole^2^ + 8.9709 × ln(Nitrogen count) + 1152.9277 × 1.0/all bond count − 71.1491. (2)

**Figure 3:**
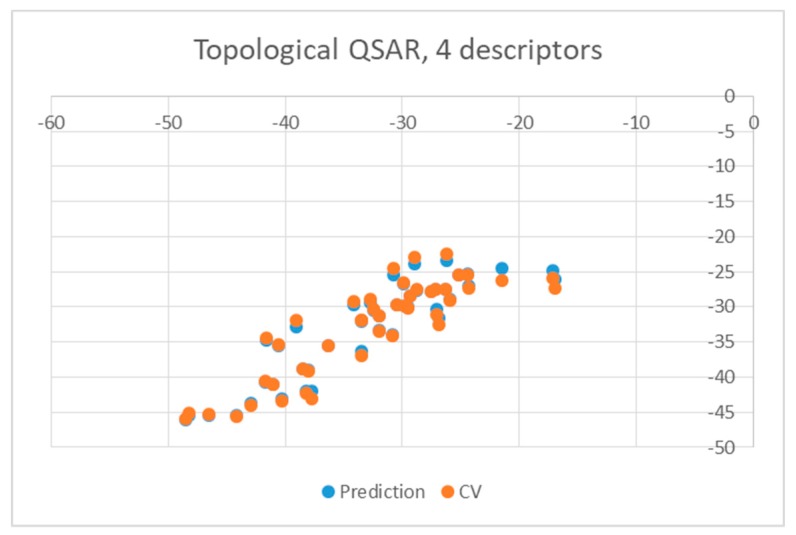
Obtained topological quantitative structure-activity relationship (QSAR) results obtained with four descriptors for wild type.

**Figure 4:**
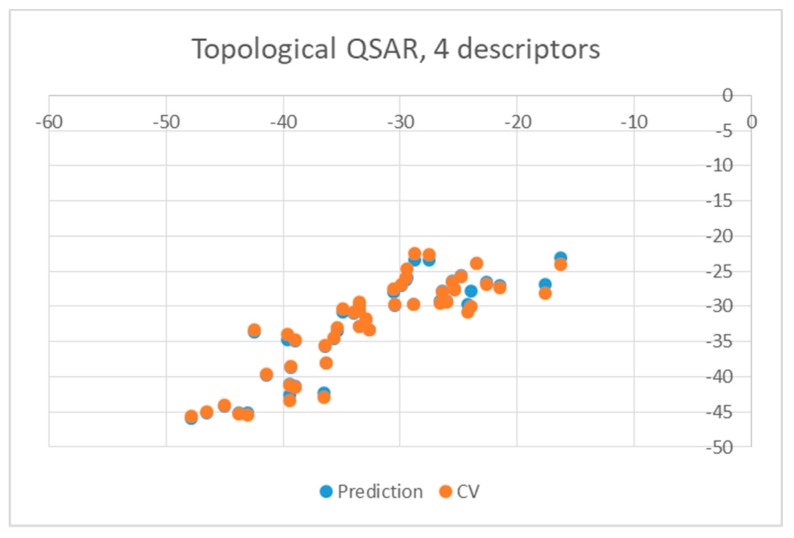
Obtained topological QSAR results obtained with four descriptors for mutants.

Both equations exhibited similar statistics characterized by an r^2^ equal to 0.8028 and 0.7817 for the wt and mutant enzyme, respectively, as well as a F-ratio of 41.7281 and 36.7059, respectively. The standard deviation in the error predicted by leave-one-out cross-validation and associated r^2^ were 3.8481 and 0.7419 for wt enzyme and 4.0614 and 0.7604 for mutants, respectively, suggesting that the equations were stable enough to be used for predictions. The quality of predictions is also illustrated in Figure 3 and Figure 4, in which the energies of binding predicted by the QSAR are plotted against the values obtained from docking (blue circles). Yellow points represent results obtained by leave-one-out cross validation (CV).

As may be seen, both relationships are very similar; an increase of numerical values of the descriptors leads to a decrease in the affinity. Relative weights of the normalized coefficients of the QSAR equations collected in Table 2 imply similar influence on the binding affinity for all four parameters. Physico-chemical interpretation of the descriptors suggests that the inhibitors should be large, uniformly hydrophobic, and they should have a small number of carboxyl groups and nitrogen atoms.

The second attempt aimed at creating a QSAR was based on a fragment contribution approach using common substructures present in the training set. For this purpose, the ADMEWORKS ModelBuilder was used [35,36]. Due to the size of the training set, sets of 6 descriptors were chosen, as in our previous work [32]. The substructures contained in both sets are shown in Table 3.

The obtained r^2^ value of less than 70% in the leave-one-out cross-validation did not encourage its use for direct prediction of the unknown compounds. Thus, it was rather the sign of the linear regression equations weight coefficients that were considered. These are a measure of the influence of a given substructure’s contribution to the overall activity; negative values indicate improvement in binding, while positive values suggest that the corresponding substructures should be eliminated or their presence minimized. The obtained results are summarized in Table 4, where substructures with the positive contribution to binding are presented on a green background, while those which should be avoided are distinguished by a red background.

**Figure 5:**
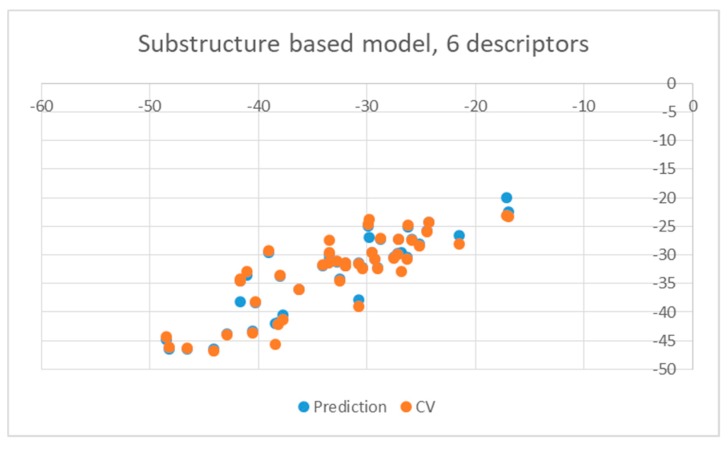
Obtained substructure-based QSAR results obtained with six descriptors for wild type.

**Figure 6:**
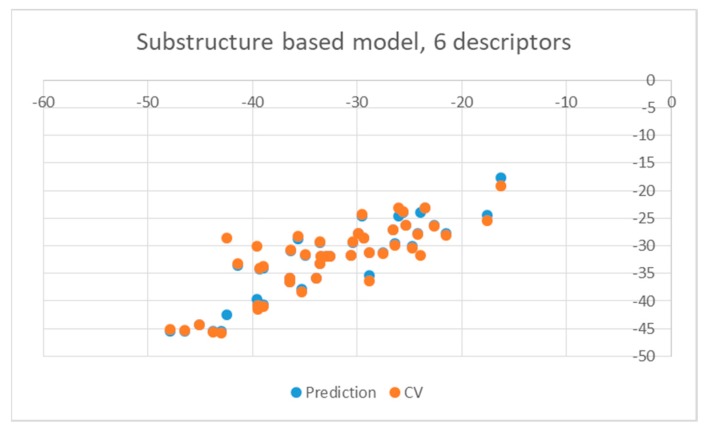
Obtained substructure-based QSAR results obtained with six descriptors for mutants.

As illustrated by Figure 5 and Figure 6, the quality of predictions is again very good. The exceptional behavior of the DJZ inhibitor, noticed in docking results, is revealed by the significant difference between the prediction based on the full set of studied compounds and that of the leave-one-out result (point at -42.5 in Figure 6), suggesting its substantial difference in the mode of action or properties from the rest of the inhibitors. Also in agreement with the descriptor-based approach was the finding that the presence of both tertiary nitrogen atoms and aromatic rings should be minimized. A strong positive component of substructure 3 that correlates with the overall size of the molecule was again in line with the conclusion obtained from the 1/bond count descriptor in the relationship obtained by SCIGRESS. Specific activity against mutations appeared to come from two specific substructures, labeled 44 and 53 in Table 3. The presence of the carboxyl groups, on the other hand, appeared to lower the binding activity of the inhibitors.

## 3. Materials and Methods

In docking, 107 structures of HIV-1 reverse transcriptase with ligands bound in the allosteric site (available from the Protein Data Bank) (www.rcsb.org) [35] were used. This included 72 structures of the wild type enzyme: 1bqm, 1c0t, 1dtq, 1dtt, 1fk9, 1hpu, 1hpz, 1ikw, 1rt2, 1rt4, 1s6p, 1s9e, 1suq, 1sv5, 1tkt, 1tkx, 1tkz, 1tl1, 1tl3, 1vrt, 1vru, 2be2, 2hnd, 2jle, 2opp, 2rf2, 2rki, 2vg5, 2vg6, 2vg7, 2wom, 2won, 2ykm, 2ykn, 2yng, 2ynh, 2yni, 2zd1, 3dle ,3dlg, 3drp, 3hvt, 3irx, 3is9, 3lak, 3lal, 3lam, 3lan, 3lp0, 3lp1, 3m8p, 3m8q, 3mec, 3mee, 3qip, 3qlh, 3qo9, 3v81, 3v81, 4b3q, 4g1q, 4i2p, 4i2q, 4i7f, 4icl, 4ko0, 4puo, 4pwd, 4q0b, 5cym, 5cyq, and 5k14, out of which three structures (4puo, 4pwd, and 4q0b) were tetramers with slightly different allosteric site architecture. For these three structures, binding of ligands to both sites was studied. Furthermore, 32 structures of the mutated RT enzyme: 1bqn, 1fko, 1fkp, 1ikv, 1jkh, 1jla, 1jlc, 1jlg, 1lw0, 1lwc, 1lwe, 1lwf, 1s1t, 1s1u, 1s1v, 1s1w, 1s1x, 2hny, 2ic3. 2opq. 2opr, 2ops, 2ynf, 2ze2, 3bgr, 3dm2, 3dmj, 3dok, 3dol, 3med, 3meg, and 5fdl were studied, bearing 12 types of mutations, with the most dominant mutation being K103N followed by Y181C. The set included six double mutations and one triple mutation. In the considered structures, 48 ligands were present. They represented a number of different classes of chemicals. By far the most frequently studied (25 entries) ligand was NVP. Codes and structures of the remaining ligands are presented in Table 5. We have excluded the AC7 ligand from the QSAR studies because its polarized surface area by far exceeded that of the other ligands. This could have potentially led to erroneous results as the allosteric cavity is hydrophobic and the QO9 ligand, for example, contains silicon atoms, for which there is no parametrization.

Docking was performed using the HYDE scoring function [37] as implemented in the LeadIT software package [38]. The receptor was prepared using the graphical interface of the package. Both protein chains were selected, and the binding site was defined to include residues within 6.5 Å radius around the native ligand. A library of ligands was imported from the mol2 file. Protonation states corresponding to the aqueous solution were used. Soft docking (allowing for volume overlap up to 100 Å^3^) was performed. For ligand base placement, the default hybrid Enthalpy and Entropy strategy was used. The clash factor was set to 0.6. Default values were used for other parameters. Scores of the top-ranked poses of each ligand were averaged for a given enzyme structure.

QSAR analysis was performed in two different modes. In both cases the training set included 46 structures, the end-point was the average docking score obtained from HYDE calculations after exclusion of results for AC7 and QO9. The size of the data set was too small to allow for splitting it into training and validation sets, so an internal leave-one-out cross-validation approach was chosen instead.

In the first approach, based on classical descriptors, several models were created using the Complete Topological QSAR method and regression equation, created by feature selection with the enhanced replacement method [ERM], as implemented in SCIGRESS Suite software [35]. In the second approach, QSAR was based on molecular fragments contribution. The common substructures were extracted from the training set. Altogether, the 96 most common fragments in all the dataset produced by ModelBuilder’s extract substructures feature were used. The six most important features for each enzyme type were selected using the particle swarm optimization algorithm, with 1000 individuals run until convergence was observed (about 3000 iterations). The six most often used were selected as descriptors. The final model was created by applying multiple linear regression.

## 4. Conclusions

The major aim of the studies presented herein was the identification of features improving activities of NNTRIs in general and in particular those specifically suited for the most common mutations of HIV-1 RT. The interpretation of the results of the fragment-based QSAR led to similar conclusions as those made for the topological QSAR: The presence of tertiary nitrogen atoms and nitriles should be avoided, while large, uniformly hydrophobic molecules are preferable. The presence of carboxyl groups appears to lower the binding affinity of the inhibitors to mutated forms of the enzyme, while the presence of moieties 44 and 53 (illustrated in Table 3) improves it.

## Figures and Tables

**Table 1:** Averaged HYDE docking scores for all ligands docked to wild type (wt) and mutated human immunodeficiency virus 1 (HIV-1) reverse transcriptase structures.

Ligand	wt	Mutants	Ligand	wt	Mutants
1BT	−34.1152	−30.4307	HBY	−38.4728	−36.3343
5DV	−36.2861	−36.4489	IL5	−17.1507	−16.2802
ETR	−29.5144	−26.6089	IB1	−28.9966	−27.5694
AAP	−27.0837	−24.194	IET	−28.7788	−29.569
AC	−8.23919	−7.69253	JLJ	−33.4534	−33.9484
ADB	−24.4548	−23.5214	KBT	−32.7463	−33.5286
BML	−41.6980	−41.3882	KRL	−16.9556	−17.6174
CXD	−29.3032	−26.394	KRP	−27.2074	−30.5539
DIZ	−26.8585	−35.3463	KRV	−25.1656	−24.7641
EFZ	−33.4729	−35.689	MRX	−24.3403	−22.6474
EUR	−30.4311	−28.8389	NNB	−31.9689	−33.4506
FPT	−27.5513	−29.8596	NNC	−30.8118	−32.6434
FTC	−26.3065	−21.4942	NNI	−33.5227	−32.9436
G73	−32.4751	−34.9394	NVE	−37.7553	−39.4643
GFA	−38.0480	−39.3134	NVP	−25.9061	−25.3768
GWB	−38.2004	−39.4685	RPV	−7.04157	−6.83753
GWE	−39.0764	−39.6241	TNK	−31.9944	−33.5303
GWI	−21.5385	−23.9688	TT1	−41.0422	−38.9757
GWJ	−26.2591	−29.3954	UCL	−40.5484	−38.9499
H12	−44.1400	−42.9899	UDR	−40.3172	−36.4783
H16	−48.2483	−46.4841	WHU	−29.9227	−45.0533
H18	−48.4723	−47.8513	YKN	−30.7327	−25.5981
H20	−46.5444	−43.7552	ZZE	−29.7661	−28.8066
HBQ	−41.6605	−42.4763	QO9	−7.04157	−6.83753

**Table 2:** Relative weights of the descriptors for wild type and mutated HIV-reverse transcriptase (-RT).

Descriptor	Normalized Coefficient (wt)	Normalized Coefficient (mutants)
carbonyl count/MW	0.4412	0.4152
hydrophobic dipole^2^	0.5324	0.4130
ln (Nitrogen count)	1.0000	1.0000
1.0/all bond count	0.7695	0.8702

**Table 3:**
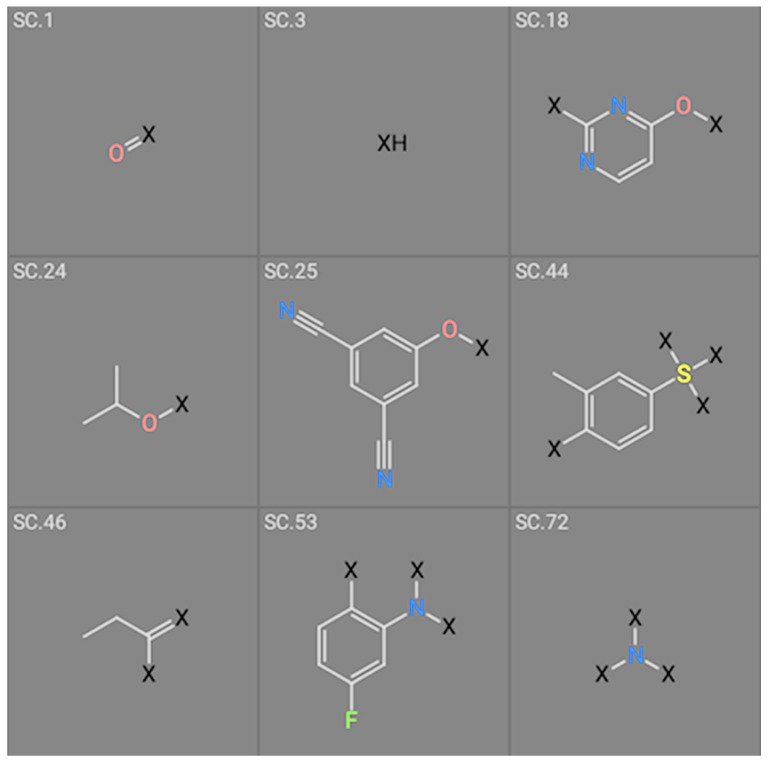
Substructures used in fragment-based QSAR for wt and mutated enzyme

**Table 4:** Weight vector normalized coefficients of the substructure-based QSAR models.

Structure #	Coefficient wt	Coefficient Mutants
1	1.84922	Not used
3	−4.12649	−4.15675
18	1.58811	Not used
24	−2.07260	Not used
25	3.688460	3.63206
44	Not used	−1.39970
46	Not used	1.13761
53	Not used	−2.04931
72	5.30721	5.31799

**Table 5:** Structures of ligands used in studies.

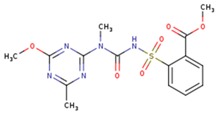	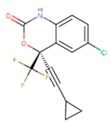	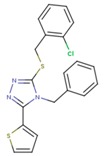
1BT	EFZ	TT1
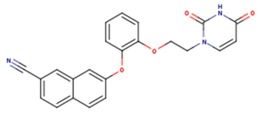	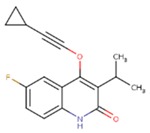	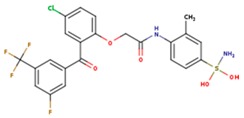
JLJ	GWB	GWE
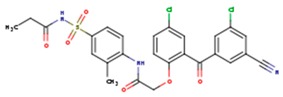	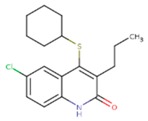	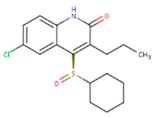
GWI	H16	H18
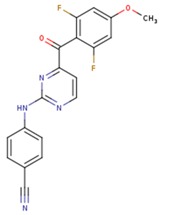	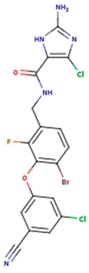	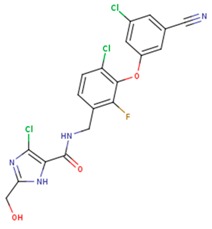
IB1	WHU	EUR
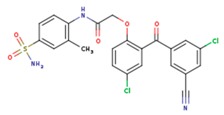	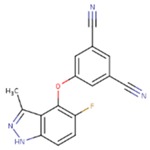	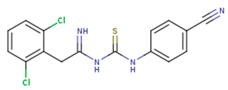
GWJ	I15	IET
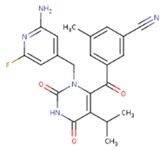	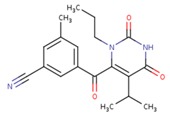	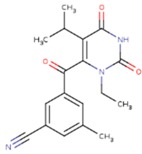
KR1	KRP	KRV
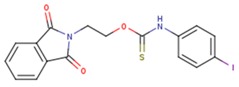	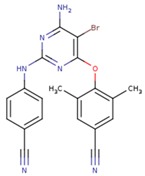	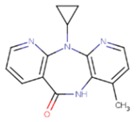
NNI	65B	NVP
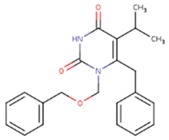	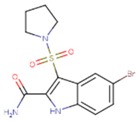	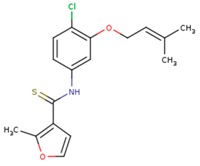
TNK	MRX	UC1
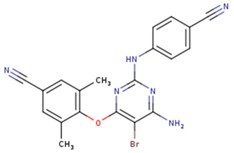	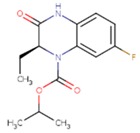	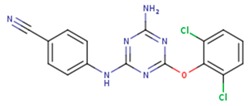
ETR(T27)	HBQ	ADB
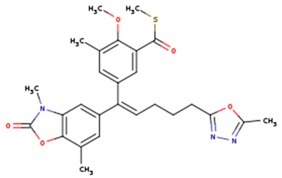	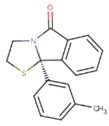	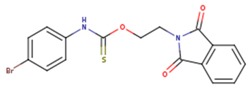
UDR	BM1	NNB
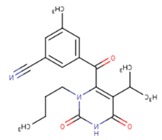	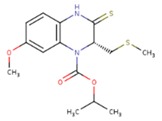	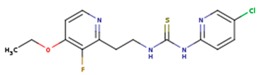
KBT	HBY	FTC
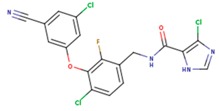	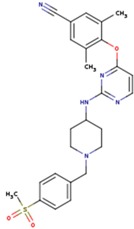	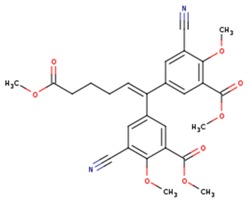
CXD	DJZ	AC7
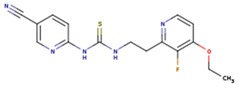	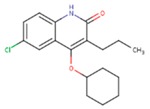	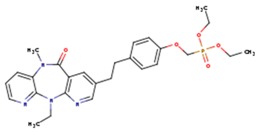
FPT	H12	NVE
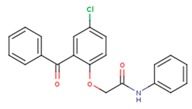	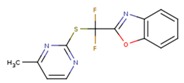	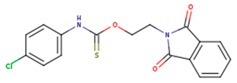
GFA	YKN	NNC
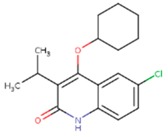	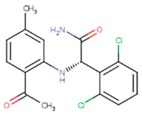	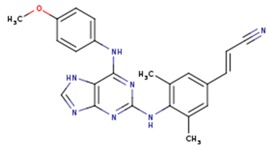
H20	AAP	G73
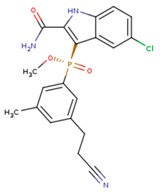	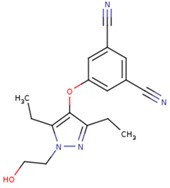	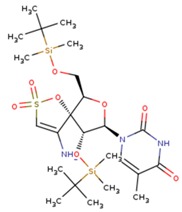
5DV	ZZE	QO9
